# Evaluation of Local Mechanical and Chemical Properties via AFM as a Tool for Understanding the Formation Mechanism of Pulsed UV Laser-Nanoinduced Patterns on Azo-Naphthalene-Based Polyimide Films

**DOI:** 10.3390/nano11030812

**Published:** 2021-03-22

**Authors:** Iuliana Stoica, Elena-Luiza Epure, Catalin-Paul Constantin, Mariana-Dana Damaceanu, Elena-Laura Ursu, Ilarion Mihaila, Ion Sava

**Affiliations:** 1 “Petru Poni” Institute of Macromolecular Chemistry, 700487 Iasi, Romania; constantin.catalin@icmpp.ro (C.-P.C.); damaceanu@icmpp.ro (M.-D.D.); ursu.laura@icmpp.ro (E.-L.U.); 2 Faculty of Chemical Engineering and Environmental Protection, “Gheorghe Asachi” Technical University, 700050 Iasi, Romania; lepure@tuiasi.ro; 3 Integrated Center of Environmental Science Studies in the North-Eastern Development Region (CERNESIM), “Alexandru Ioan Cuza” University of Iasi, 700506 Iasi, Romania; ilarion.mihaila@gmail.com

**Keywords:** azo-polyimide, surface relief grating, AFM PinPoint, topographical analysis, nanomechanical characterization, molecular simulation

## Abstract

Aromatic polyimides containing side azo-naphthalene groups have been investigated regarding their capacity of generating surface relief gratings (SRGs) under pulsed UV laser irradiation through phase masks, using different fluencies and pulse numbers. The process of the material photo-fluidization and the supramolecular re-organization of the surface were investigated using atomic force microscopy (AFM). At first, an AFM nanoscale topographical analysis of the induced SRGs was performed in terms of morphology and tridimensional amplitude, spatial, hybrid, and functional parameters. Afterward, a nanomechanical characterization of SRGs using an advanced method, namely, AFM PinPoint mode, was performed, where the quantitative nanomechanical properties (i.e., modulus, adhesion, deformation) of the nanostructured azo-polyimide surfaces were acquired with a highly correlated topographic registration. This method proved to be very effective in understanding the formation mechanism of the surface modulations during pulsed UV laser irradiation. Additionally to AFM investigations, confocal Raman measurements and molecular simulations were performed to provide information about structured azo-polyimide chemical composition and macromolecular conformation induced by laser irradiation.

## 1. Introduction

Polyimides (PIs) represent an important class of high-performance polymers that are exploited in a variety of applications due to their excellent physicochemical properties such as optical and thermal stability in combination with high glass transition temperature, low susceptibility to laser damage, and low dielectric constant value [1,2]. Moreover, polyimides have been investigated as potential materials in the fields of optoelectronics and photonics [3]. Particularly interesting are polyimides containing azobenzene units, which have already been investigated for photoinduced alignment in liquid crystal display [4], as photomechanical response materials [5], and for holographic diffraction grating recording [6,7,8,9,10,11]. The azo derivatives can be introduced into polymers in different ways: by polymerization reaction between monomers, which contain preformed azobenzene group (diamines or dianhydrides); by dissolving a guest chromophore in a polymer matrix; by attaching the chromophores covalently to the polymer chains; or by using of different nonconvalent intermolecular interactions (ionic interactions, coordination bonds, hydrogen bonds, π–π interactions) between the dye and the polymer backbone. The first method is the most used due to the better control of sequence chromophore distribution. The azobenzene-functionalized monomers can be the diamines or dianhydrides or both of them [3,12,13,14,15,16]. Many azodiamines have been obtained and used for the synthesis and characterization of the azopolyimides, usually containing substituted or unsubstituted azobenzene side chains. Thus, a broad range of azopolyimides, azo polyamide-imides, or azo polyester-imides have been published thus far [12,15,17,18,19]. The majority of polymers functionalized with azobenzene derivatives under the action of linearly polarized light undergo multiple reversible *trans* to *cis* photo-isomerization processes. A supramolecular organization process is generated as a result of the perpendicular alignment of the azobenzene molecules to the electric field vector, controlling the photo-induced optical anisotropy. Thus, the azo-polymer photo-fluidization due to the above-described phenomenon can appear in the exposed regions [20,21]. Moreover, the cyclic photo-isomerization can lead to a large-scale mass transport of the polymer chains, which appear as a surface relief grating (SRG) [13,14,22,23]. Mainly, this mass displacement can take place from UV laser-exposed areas to the unexposed areas. Recently, it was demonstrated that also an inverse mass displacement can occur, from the dark regions to the illuminated ones [24]. The arguments for the development of the azobenzene photoisomerization process by inversion or rotation are the subject of numerous molecular simulation studies [25,26,27,28]. A large number of articles track the computational characterization of these photocontrolled materials due to their prospective applications in different fields such as liquid crystals [29,30,31], optical data storage [32], photosensitive micelles [33], triggers in protein folding [34], and so on. Due to the complexity of the molecular migration phenomenon that induces the SRG formation process, the number of molecular studies is significantly lower [35,36,37].

Over time, in order to obtain well-defined and stable SRGs on azo-polymers, researchers have applied two main techniques, namely, pulsed and continuous UV laser irradiation. In the case of continuous laser irradiation, the apexes of the sinusoidal surface relief appeared in the unexposed regions. When pulsed UV laser irradiation was employed, the positions of the apexes were dependent on the laser fluence, being in the exposed regions when the fluence value was below a threshold, and in the unexposed regions when the fluence value was above this threshold, as mentioned in the literature data [24]. In a pulsed laser regime, the relief geometric parameters depend on several factors. The design of the high-quality phase masks and their characteristics (the material used in fabrication, the fabrication method, the type of the lattice grating structures, the number of pitches, the period of the lattice, the period accuracy and uniformity, the efficiency (%) of the phase masks) are mainly responsible for the resulted pattern aspect. The nanostructures can also be strongly affected by the experimental parameters of the pulsed laser irradiation method, such as the incident laser wavelength, the incident laser fluence, the polarization of the laser, the angle of incidence, the number of laser pulses, and the duration of one laser pulse. Last but not least, the generation of the SRGs is dependent of the chemical structure of the azopolymer, in terms of the backbone flexibility and position of the azo-group in the polymer chain (inducing certain architecture), the pristine film surface morphology subjected to pulsed laser nanostructuring, and the film thickness.

Many researchers have been focused on the progress in atomic force microscopy (AFM) techniques to characterize the polymer films at the nano-scale by recording the local mechanical behavior, especially the force response to approach, even simultaneously with the recording of the morphology [38,39]. Over the years, the progress of several research groups in data acquisition and signal processing has allowed manufacturers to develop this technique, under different names, such as pulsed force mode (PFM), tested by WiTec company (Ulm, Germany) for scanning force microscopy (SFM) systems from CSEM, Digital Instruments, Molecular Imaging, Park, Seiko and TopoMetrix [40], PeakForce Quantitative Nanomechanical Mapping (PeakForce QNMTM, referred to as QNM for brevity, by Bruker), HybriD Mode Atomic Force Microscopy from NT-MDT (Zelenograd, Moscow, Russia) [41,42,43], Quantitative Imaging (QI, by JPK Instruments), or PinPoint mode from Park Systems Corp. (Suwon, Korea) [44]. Each has different features. Fast force spectroscopy mapping via PinPoint mode was designed to prevent positional errors by simultaneously acquiring accurate height and force–distance information in the whole scanning area, while the cantilever tip is lifted at every pixel at a perpendicular angle from the sample surface.

In this context, our study focused on the investigation of a newly synthesized aromatic polyimide containing azo-naphthalene side groups with the aim to evaluate the local morphological, mechanical, and chemical properties via atomic force microscopy, especially in PinPoint mode, and confocal Raman spectroscopy. It is meant to provide a deep understanding of the formation mechanism of SRGs induced by pulsed UV laser on these azo-polyimide films, through phase masks, using different fluencies and number of pulses. Moreover, this article attempts to correlate the macroscale behavior of this azo-naphthalene-based polyimide during photoisomerization with the one from the atomic level simulating certain polymeric systems with a different content of the *cis* isomer. The application of quantitative nanomechanical properties (i.e., modulus, adhesion, deformation) acquired with highly correlated topographic registration using PinPoint mode to describe the formation of nanostructured azo-naphthalene-based polyimide surfaces has not been reported yet in the literature to the best of our knowledge. The molecular simulation and confocal Raman spectroscopy complete this study, which promises to significantly advance the research in this field beyond the state of the art.

## 2. Materials and Methods

### 2.1. Material

**Figure 1:**
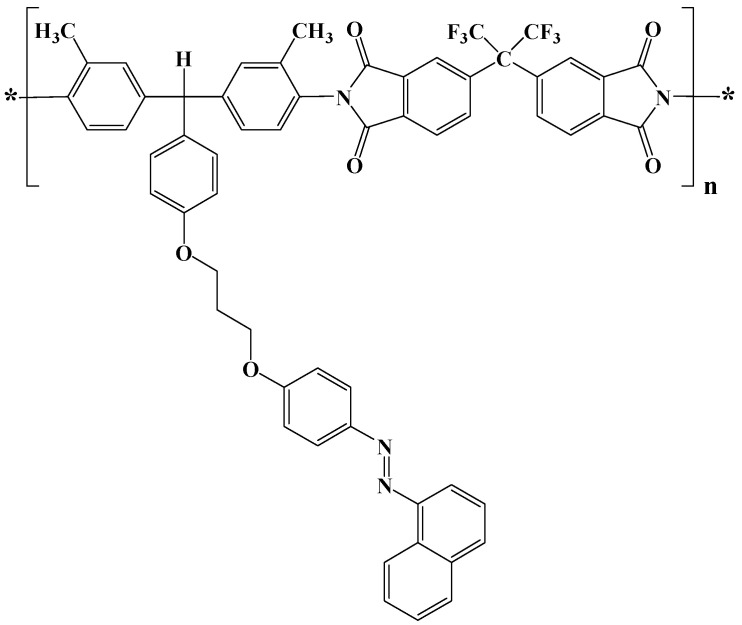
Chemical structure of the novel azo-naphthalene-based polyimide, AzoPI, used in this study.

An aromatic diamine containing the azo group (-N=N-) pendent to the triphenylmethane core was synthesized by the Williamson reaction of 4-[bis-(4-amino-3-methyl-phenyl)-methyl]-phenol with 3-[4-naphthalen-1-ylazo)-phenoxy]-bromopropane. Details regarding the preparation of this azodiamine and the corresponding intermediates have been recently reported elsewhere [45]. 4,4′(Hexafluoroisopropylidene)diphthalic anhydride (6FDA) was purchased from Aldrich and used as received. The azopolyimide has been synthesized by the polycondensation reaction of 6FDA with the above mentioned azodiamine by using a procedure previously reported [12,45]. The structure of the azo-naphthalene-based polyimide is shown in Figure 1. The thickness of the azo-naphthalene-based polyimide films, measured using a profilometer, was 3094–3117 nm, the average value being 3.10 ± 0.01 μm.

As previously reported in [45], the irradiation with UV light of 365 nm will induce the photoisomerization of the *trans* isomer to the *cis* isomer. The intensity of the absorption bands located at 384 nm (due to the π → π* transitions in the *trans* isomer) decreased progressively as the irradiation time advanced, being assisted by the increase of the absorption band corresponding to the π → π* and n → π* transitions in the *cis* isomer, located at approximately 290 and 472 nm, respectively. Moreover, the corresponding isosbestic points were found at 325−326 and 450−459 nm, and the absorption coefficient was 1.1143 × 10^4^ cm^−1^. The absorption coefficient has been calculated by using the equation: k = 2.303 A/d, where A is the absorbance and d is the thickness of the sample. Futhermore, the detailed evaluation of *cis*–*trans* thermal isomerization was carried out by UV–VIS spectroscopy both in solution and in solid state for the starting azodiamine monomer and corresponding azopolyimide, as it was largely presented in reference [45]. The thermal relaxation induced the total recovery of *trans* isomer after 2700 s. The thermal relaxation was reflected in the gradual increase of the bands at 384 nm (π → π* transitions, *trans* isomer) and the progressive decrease of the bands at 290 nm (π → π*) transitions, *cis* isomer) and 472 nm (n → π* transitions, *cis* isomer) during exposure at 70 °C for different intervals of time.

### 2.2. Molecular Simulations

The molecular dynamics simulations were performed with Materials Studio 4.0 software [46]. First, the structural units corresponding to the *trans* and *cis* isomers were built. These structural units were minimized through ab initio calculations within the DMol^3^ module using the Perdew-Wang PWC functional until fulfilling a 2 × 10^−5^ Hartree energy convergence. Subsequently, 3 polymer chains of 10 structural units each were built, with different content of *cis* isomer: AzoPI (with 15% *cis* groups), AzoPI_50%*cis* (with 50% *cis* groups), and AzoPI_100%*cis* (with 100% *cis* groups). The *cis* azo units were randomly distributed along the polymer chain. Both the structural units and the polymers were minimized with the Forcite module using the Dreiding force field. Three-dimensional amorphous structures were generated using the Amorphous Cell module. Each cell was built to contain three polymer chains of the same kind, at a density of 1 g/cm^3^, at 298 K. The final structures were obtained according to the following protocol: (step 1) minimization (energy convergence 1 × 10^−4^ kcal/mol), (step 2) 2 anneal cycles that were driven in the range of 300–800 K for 40 ps, (step 3) compression/constriction the cell by molecular dynamics (NPT ensemble (constant number (N), volume (V), and temperature (T); T is regulated via a thermostat; pressure (P) is regulated), T = 298 K, Berendsen thermostat and barostat) until the density reaches a value around 1.2 g/cm^3^, (step 4) equilibration of the cell through an NVT dynamics (constant number (N), volume (V), and temperature (T); T is regulated via a thermostat, pressure (P) is unregulated) for 500 ps (T = 298K, Nose thermostat). Achieving constant values for the density-time curves in NPT stage and temperature/energy-time in NVT-MD stage indicates that the system is stable and has reached an equilibrium value. Data collection was performed after another 200 ps of dynamic simulations in NVE ensemble (constant number (N), volume (V), and energy (E); T is regulated via a thermostat, pressure (P) is unregulated), at T = 298 K.

### 2.3. Laser Patterning

**Figure 2:**
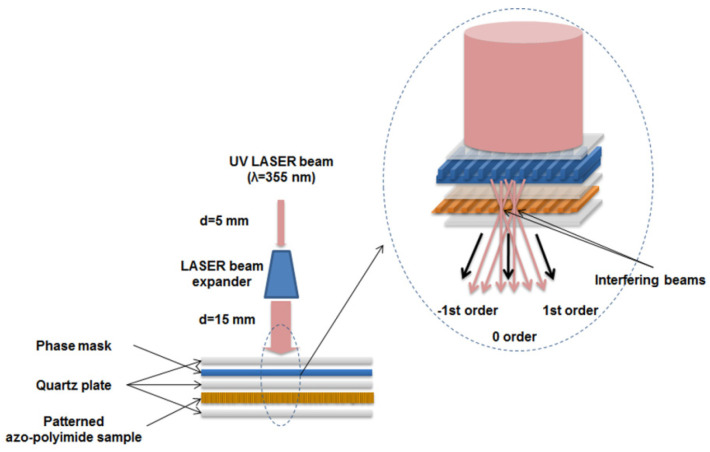
Pulsed laser irradiation through a phase mask setting, with a detail on the formation of interference image on the azo-naphthalene-based polyimide surface.

In order to induce micro/nano structuration on the azo-polyimide surface, we used the setting presented in Figure 2. A pulsed Nd:YAG laser (Brilliant B from Quantel) working at third harmonic (355 nm wavelength), with a diameter of 5 mm. The laser was horizontally polarized (*s*-polarized), meaning that its light oscillated along a horizontal plane. The electric field vector was perpendicular to the plane of incidence, to the direction of gravity, and to the direction of light propagation. In this way, the grating region was uniformly illuminated. This choice can be motivated on the basis of observations of Miniewicz and collaborators [47], according to which the grating strength in the case of *s*-*p* inscription attains higher values. The pulse duration of 6 ns was chosen. The pulse repetition rate was 10Hz. The aperture of 5 mm laser beam was enlarged until 15 mm, by placing a beam expander with a fixed ratio of 3×. A diffraction phase mask (Edmund Scientific Co., Barrington, NJ, USA) with a thickness of 76 μm and 1000 grooves per mm was used. The linear diffraction grating period was about 1 μm. After passing through the diffractive optical element, the laser beam generates an interference field in its proximity, leading to a laser interference pattern on the azo-polyimide surface, placed after the phase mask, producing features with the pitch of the same order of magnitude. The quartz plate placed between the phase mask and the sample had a thickness of 1 mm. Two incident fluences, namely, 10 mJ/cm^2^ and 45 mJ/cm^2^ (measured after beam expander), and two different numbers of pulses, namely, 10 and 100, were used. Therefore, the samples were named using the label AzoPI i/j, where i is the incident fluence and j is the number of irradiation pulses, as follows: AzoPI 10/10, AzoPI 10/100, AzoPI 45/10, and AzoPI 45/100.

### 2.4. Measurements

The AFM investigations on the nanostructured azo-polyimide films were performed in semi-contact mode, in the atmospheric conditions, at room temperature, on a surface area of 10 × 10 µm^2^, using a Scanning Probe Microscope Solver Pro-M from NT-MDT, Russia, using a high-resolution “golden” silicon AFM probe NSG01 (NT-MDT, Zelenograd, Moscow, Russia) with a typical curvature radius of 10 nm and the free resonant frequency of 90.5 kHz. AFM data acquisition and analysis were performed using Nova software from NT-DMT. The tridimensional parameters were calculated using Image Analysis 3.5.0.19892 software. Nanomechanical measurements were made on a Park NX10 Atomic Force Microscope (Park Systems Corp., Suwon, Korea), using PinPoint Nanomechanical mode, which allowed us to obtain sample’s stiffness mapping through force–distance curves acquired at each pixel over the entire scanning area (of 1 × 5 µm^2^ in our case). Thus, the quantitative nanomechanical properties (i.e., modulus, adhesion, deformation) of the nanostructured azo-polyimide surfaces were acquired with highly correlated topographic registration. In order to provide the most accurate mechanical properties data, we carefully selected the Force Modulation Mode - Reflex Coating (FMR) probe (Park Systems Corp., Suwon, Korea) with a force constant of 2.8 N/m and resonance frequency of 75 kHz according to the relative stiffness of their cantilever when compared to that of the sample, so that it can offer immediate response to any changes on the surface’s material properties. The scanning frequency was 0.5 Hz, and the scanning speed was 5.0 µm/s. Besides AFM investigations, supplementary confocal Raman measurements (laser source: 632.8 nm, 50 mW; CCD detector) performed using inVia Renishaw Raman confocal microscope (Renishaw, UK) were used to provide information about structured azo-polyimide chemical composition and macromolecular conformation. Spectra were recorded in backscattering geometry using a 50× objective. Spectral manipulations such as baseline adjustment, smoothing, and normalization were performed with the WiRE 3.3 software (Renishaw, UK).

## 3. Results and Discussion

Imaging the distribution of local mechanical properties at the nanoscale can significantly advance research on exciting soft materials relevant for optoelectronic applications [48]. In our case, the SRGs were produced using phase masks through pulsed laser irradiation, with a maximum light fluency lower than the ablation threshold of the material. This method employs a diffractive optical element (phase mask) for precise spatial modulation of the UV laser writing beam. In order to obtain the interference pattern, only the zero (0) and plus and minus first diffraction orders (±1) were considered. When a UV laser beam was incident on the phase mask, the zero-order diffracted beam was minimized. Furthermore, the plus and minus first diffracted orders were enlarged as much as possible [49]. Because of the very short irradiation times (6 nanoseconds) used in the pulsed mode, the mechanism responsible for the periodic SRGs profile generation involves the fringe pattern produced by the interference of the diffracted beams. They act through two possible mechanisms, namely, a very fast supramolecular reorganization process, induced by the azo-groups dipole orientation [50], and material photo-fluidization, induced by the *trans–cis* isomerization process of the azo-segments in the exposed regions [20,24,51]. This competes with the larger proper volume required by the *cis* conformation [52]. None of the models concerning the azo-polymers surface nanostructuration mechanisms proposed until now completely explain the surface relief formation, although a consensus has been reached—the azonaftalene dipoles orientation probably induce a more organized and compacted structure, having as a result a material contraction in the light-exposed regions with the generation of grooves in the dark areas [22]. Thus, it is considered that the highest intensity of the interference pattern corresponds to the valley of SRGs, due to the mass displacement from exposed to unexposed regions, and inverse mass displacement, from uncovered to covered regions [20,24,53,54]. On the other hand, it should be mentioned that the assignment highest intensity of the interference pattern corresponds to the valley of SRGs is material-dependent and cannot be ascertained from the data presented in this paper, but was previously demonstrated by direct observations of Hurduc and collaborators 20] who proposed a mechanism for SRG formation during laser irradiation involving at least three processes: (1) the polymer photofluidization in illuminated regions, (2) the mass displacement from illuminated to dark regions, and (3) the inverse mass displacement from dark to illuminated regions, on the basis of the performed amplitude modulation-frequency modulation atomic force microscopy (AM-FM AFM) viscoelastic mapping before, during, and after light irradiation.

### 3.1. AFM Nanoscale Morphological Analysis

**Figure 3:**
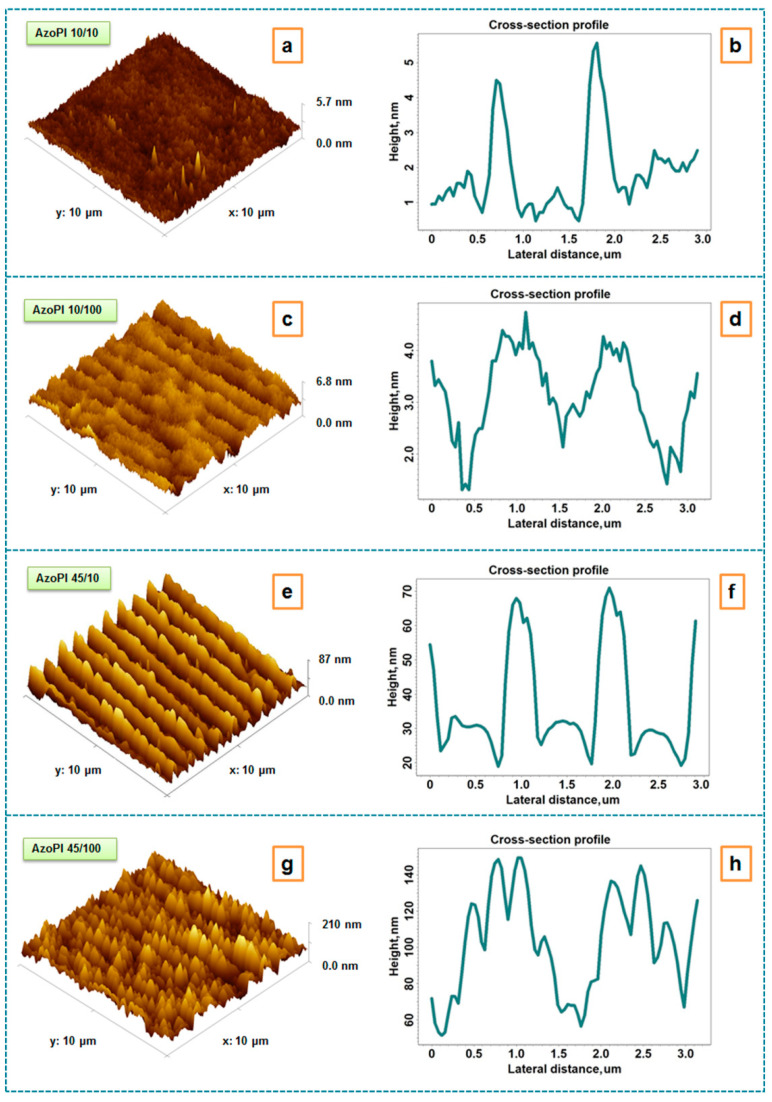
Height 3D atomic force microscopy (AFM) images and corresponding cross-section profiles for azo-polyimide irradiated using different laser energy density/number of pulses of irradiation: 10 mJ/cm^2^/10 pulses (**a**,**b**), 10 mJ/cm^2^/100 pulses (**c**,**d**), 45 mJ/cm^2^/10 pulses (**e**,**f**), 45 mJ/cm^2^/100 pulses (**g**,**h**).

**Figure 4:**
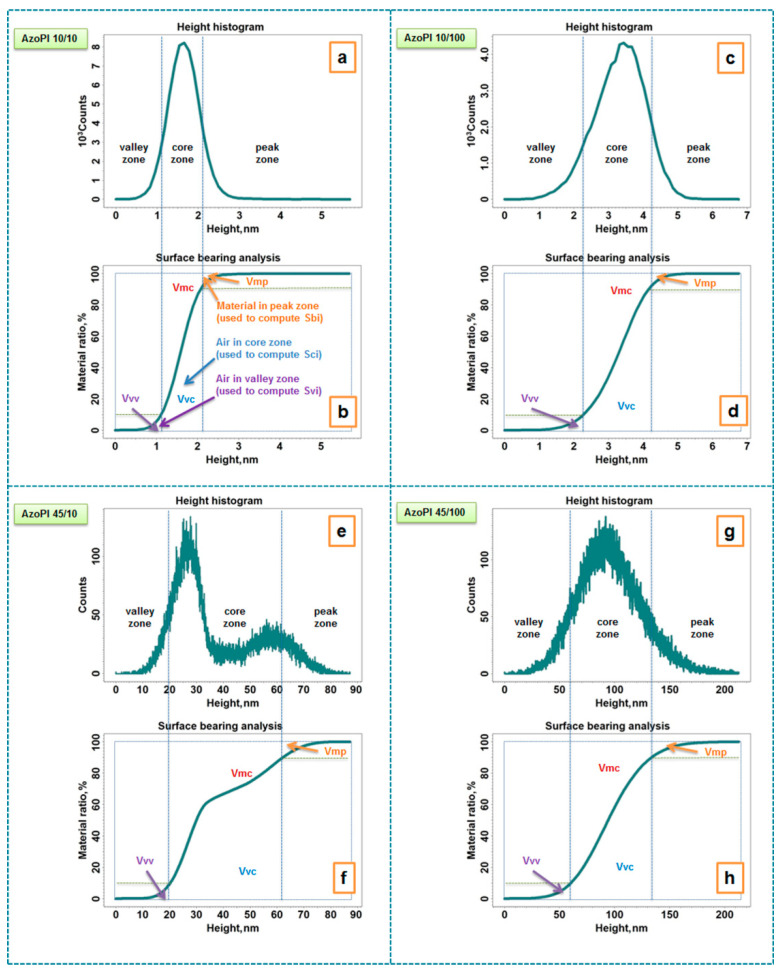
Height histograms and surface bearing area ratio curves corresponding to height AFM images for azo-polyimide irradiated using different laser energy density/number of pulses of irradiation: 10 mJ/cm^2^/10 pulses (**a**,**b**), 10 mJ/cm^2^/100 pulses (**c**,**d**), 45 mJ/cm^2^/10 pulses (**e**,**f**), 45 mJ/cm^2^/100 pulses (**g**,**h**).

The occurrence of the SRGs induced by the UV laser irradiation on azo-polyimide surface was highlighted using AFM investigations. Figure 3 presents the height 3D AFM images and the corresponding cross-section profiles obtained for the azo-polyimide films irradiated with different laser energy density/number of pulses of irradiation, as follows: 10 mJ/cm^2^/10 pulses (a,b), 10 mJ/cm^2^/100 pulses (c,d), 45 mJ/cm^2^/10 pulses (e,f), 45 mJ/cm^2^/100 pulses (g,h). According to the height AFM images, Figure 4 depicts the height histograms and surface bearing area ratio curves of the azo-polyimide films irradiated using different laser energy density/number of pulses of irradiation: 10 mJ/cm^2^/10 pulses (a,b), 10 mJ/cm^2^/100 pulses (c,d), 45 mJ/cm^2^/10 pulses (e,f), 45 mJ/cm^2^/100 pulses (g,h).

The anchoring of the azo group through an aliphatic spacer on the polyimide chains with flexible conformations given by the presence of hexafluoroisopropylidene groups induced a high isomerization/structuring capacity, transposed in the generation of repetitive surface structures through the phase mask, even at low energies and a small number of pulses. As can be seen in Figure 3a,b, from the AFM 3D image and the corresponding cross-section profile, by using an energy density of 10 mJ/cm^2^ and 10 pulses of irradiation, we found that the SRGs were not so visible, being only a few nanometers high. At this low energy, as the number of pulses increased, the structures became wider and better highlighted, although their height did not differ much from those obtained by using the small number of pulses (Figure 3c,d). Analyzing the profile diagram of the structured sample with higher energy density of 45 mJ/cm^2^ and using 10 pulses of irradiation, we observed a very good uniformity of the SRGs’ appearance, with the amplitude of the modulation being ≈50 nm (Figure 3e,f). This was due to the great mobility of the azo group in the side chain and flexibility of the main chain, as well as the existing free volume. Consequently, the polymer responded better to a high energy density of 45 mJ/cm^2^ and a small number of pulses (10). Moreover, it can be observed that in this case the SRGs were narrow, as in the previous case, when a small number of irradiation pulses were used. As the energy was increased, the repetitive structures became more and more defined, increasing in height with the increase of the number of pulses until 100 nm (Figure 3g,h) as observed from the cross-section profiles. It seemed that from the structural uniformity point of view, the irradiation with high energy and small number of pulses was the most indicated. At large number of pulses (100), their aspect was a corrugated one, as can be seen from the cross-section profile (Figure 3g). The obtained image suggests the occurrence of possible phenomena that may be due to surface reorganization mechanisms (Figure 3h) or to additional nanostructuring of the formations. This complex high-amplitude morphology may be an indication that the structured sample acted itself as a diffractive element—it generated further diffraction orders that gave shorter periods (higher harmonics) to the holographic irradiation pattern. The response of the material after irradiation may have been due to the mechanisms of reorganization on the surface or induced by the appearance of the photo-fluidization state. Photo-fluidization can become significant, especially when using a large number of pulses. However, regardless of the mechanism of the surface organization, due to the conformational changes that occur in the azo-polyimide during irradiation, it is difficult to assess the accurate response to the phase mask laser irradiation process of this material. In addition, according to Viswanathan et al. [55], since mechanical forces may also act in the bulk of the film, it is possible that the orientation grating extends also throughout the thickness of the material.

The values obtained for the root mean square roughness (Table 1) were influenced by the SRGs aspect and amplitude, following the same trend. In this way, it was observed that Sq increased as the energy/number of pulses increased.

The aspect of the height histograms (Figure 4a,c,e,g) also describes the surface features created by the phase mask UV laser irradiation by means surface skewness (Ssk) and coefficient of kurtosis (Sku) (Table 1), indicating also the distribution of the relief on areas of interest: the valley zone, the core zone, and the peak zone. Details regarding their meaning can be found in Appendix A.

The hybrid parameter surface area ratio (Sdr), calculated as the ratio between the area of the real developed surface and the area of the projected surface, can be used to describe the complexity of the surface. This parameter has an important role in controlling the surface properties of the materials envisaged for use in electronics [56], being a key factor for measuring the performance. Analyzing the data from Table 1, one can conclude that the surface complexity increased with the increase of the laser energy density due to the appearance of well-defined SRGs. The most complex surface was obtained for AzoPI 45/100 sample, induced by the supplementary nanostructurations of the SRGs formations, visible in Figure 3g,h.

The values of the spatial parameter surface texture direction index (Stdi) (displayed in Table 1 and calculated examining the 3D AFM height images and the corresponding angular spectra) were indicators of whether or not a surface has a preferential orientation of its features, thus denoting the anisotropy/isotropy of the morphology. Stdi close to 1 indicates that the sample surface is isotropic, with a random surface texture that does not have any texture that stands out, with no preferential orientation and presenting identical characteristics regardless of the direction of measurement. Meanwhile, Stdi close to zero shows a dominant direction of the surface morphology [14,57]. This can indicate an oriented surface or a periodic structure. In this case, the surface is considered to be anisotropic. Therefore, for all nanostructured samples, the values of about 0.2–0.4 attributed of this spatial parameter denote the anisotropy of the morphology induced by the oriented SRGs under pulsed UV laser irradiation. Moreover, AzoPI 45/10 sample shows the highest degree of orientation and organization, indicated by the lowest value of Stdi, namely, 0.193. This information is very important in describing the anisotropy of the morphology, used in electronic applications, where the controlling of the alignment is necessary.

Upon computation by inversion of the cumulative height distribution histograms, division into zones (Figure 4a,c,e,g), the surface bearing area ratio curves or Abbott curves (Figure 4b,d,f,h) were used as the basis for calculating the functional indexes (Sbi, Sci, Svi) and functional volume parameter (Vmp, Vmc, Vvc, Vvv) [58].

These parameters, presented in Table 1, are of great importance for the pursued applications. Low surface bearing index values (Sbi < 0.608) [57] obtained for AzoPI 10/10, AzoPI 10/100, and AzoPI 45/100 films revealed surfaces with low bearing capacity. This fact is also supported by low values obtained for the peak material volume. Instead, AzoPI 45/10 sample has bearing index higher than 0.608, indicating a good bearing capacity. In the core zone, Sci is sensitive to both occasional high peaks and occasional deep valleys. AzoPI 10/10 and AzoPI 10/100 have a low core fluid retention index (Sci < 1.56) [57], induced also by the low values of the core material and void volumes, while AzoPI 45/10 and AzoPI 45/100, due to their complex morphology and high core material and void volumes, have a high core fluid retention index (Sci > 1.56) [57]. The analyzed surfaces with relatively few deep valleys have low valley fluid retention index (Svi < 0.11) [57] due to low valley void volumes.

In this way, the pattern can be designed by controlling the experimental parameters, and by calculating these parameters that describe the obtained morphology, we can select a certain pattern for a special desired electronics manufacturing application.

The topography does not provide the difference in mechanical properties (such as elasticity, adhesion). AFM phase imaging can provide mechanical properties distribution using qualitative contrast. On the other hand, force–distance spectroscopy records mechanical data quantitatively by indenting a cantilever tip on the sample surface one point at a time. In our case, the analysis of the mechanical characteristics of the newly formed nanogrooves required to acquire the distribution image and the quantitative data simultaneously.

### 3.2. Nanomechanical Characterization Using AFM PinPoint Mode

**Figure 5:**
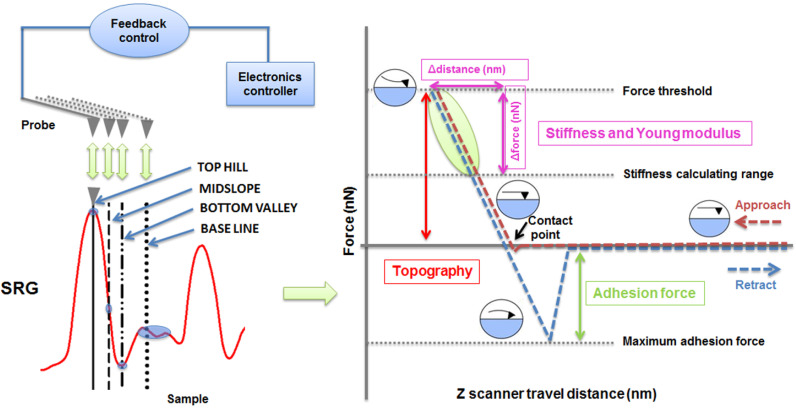
Working mechanism of PinPoint Nanomechanical mode: the tip of the cantilever is moved at each of 256 × 256 points along a sample’s surface, and the feedback system controls the approach and retraction of a probe, allowing the acquirer of both surface topography and force–distance curves, and further to extract the mechanical property data.

PinPoint Nanomechanical mode was designed to prevent positional errors by simultaneously acquiring accurate height and force–distance information in each of the 256 × 256 pixels in the whole scanning area, thus preventing the occurrence of the artifacts and positional errors in force–distance spectroscopy and topographical data. In Figure 5 and Appendix B, we present the working mechanism of PinPoint Nanomechanical mode, describing the generation of the map of the sample’s stiffness/elasticity and deformation depth from the surface concomitantly obtained through the adhesion force map with the topographic sample information [59,60,61,62,63,64].

**Figure 6:**
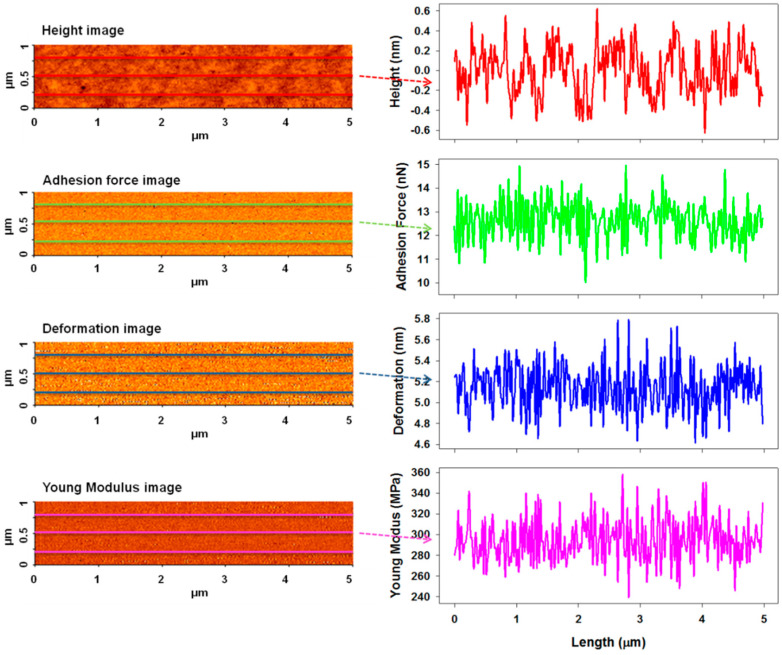
AFM PinPoint combined height, adhesion force, deformation, and Young’s modulus images and representative cross-section profiles taken along the middle line obtained for pristine azo-polyimide.

**Figure 7:**
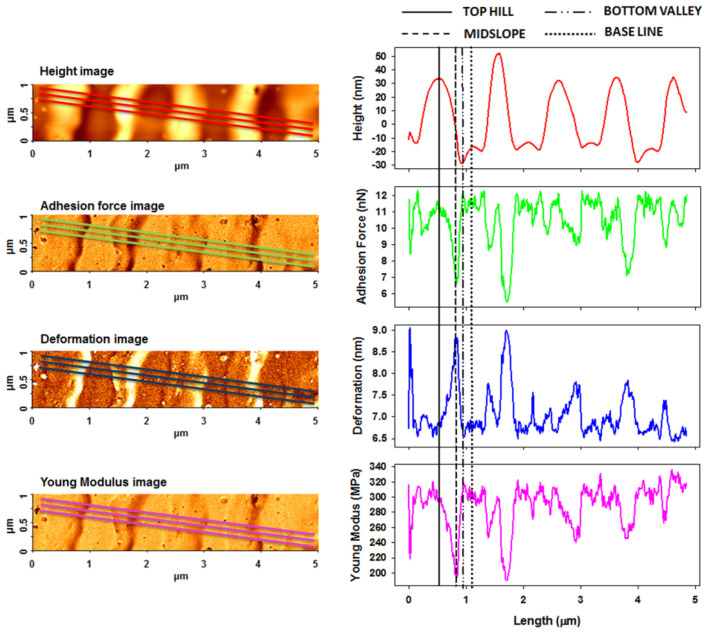
AFM PinPoint combined height, adhesion force, deformation, and Young’s modulus AFM images and corresponding cross-section profiles mediated from the presented three lines obtained for azo-polyimide irradiated with a laser energy density of 45 mJ/cm^2^ and 10 pulses.

**Figure 8:**
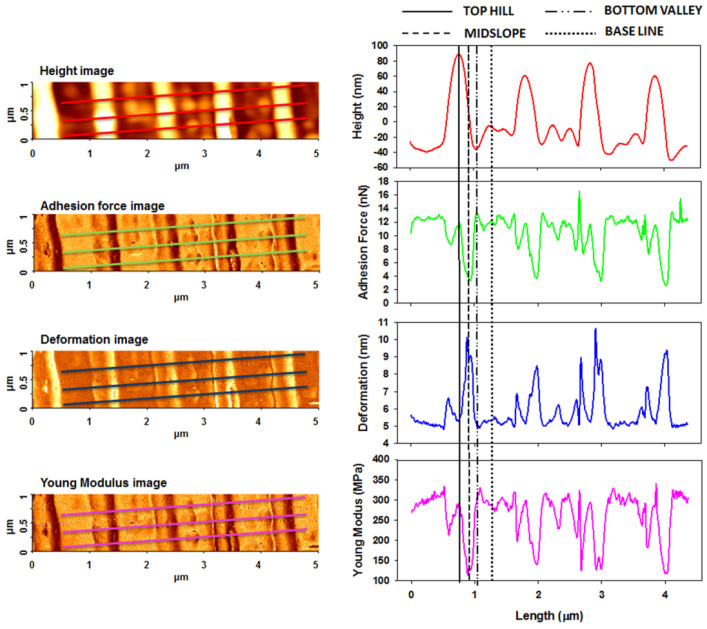
AFM PinPoint combined height, adhesion force, deformation, and Young’s modulus images and corresponding cross-section profiles mediated from the presented three lines obtained for azo-polyimide irradiated with a laser energy density of 45 mJ/cm^2^ and 100 pulses.

Figure 6, Figure 7 and Figure 8 show the PinPoint combined height, adhesion force, deformation, and Young’s modulus AFM images and corresponding cross-section profiles in the case of pristine (Figure 6) and irradiated azo-polyimide with a laser energy density of 45 mJ/cm^2^ and either 10 pulses (Figure 7) or 100 pulses (Figure 8). The last two samples were selected for this kind of measurement because the generated modulations were very well defined, facilitating the investigations in different positions of interest (on the top hills, middle slopes, base, and bottom valleys of the SRG, as indicated in Figure 5). Each profile was an average of the profiles in the band highlighted in the images, excepting the case of the pristine sample, where the surface was random.

As seen in Table 2, the pristine AzoPI presented an average adhesion force of 12.6 ± 0.8 nN, deformation of 5.1 ± 0.2 nm, and Young’s modulus of 294.8 ± 20.5 MPa. After the SRG formation as a result of pulsed UV laser irradiation, these nanomechanical characteristics were found to be different, depending on the region of the modulation where the measurements were made, namely, on SRG top hills, middle slope, bottom valleys, and baseline, as indicated in Figure 5. Thus, various values of nanomechanical parameters were obtained in these different regions, mostly induced by the density of the material, but not limited to it.

**Figure 9:**
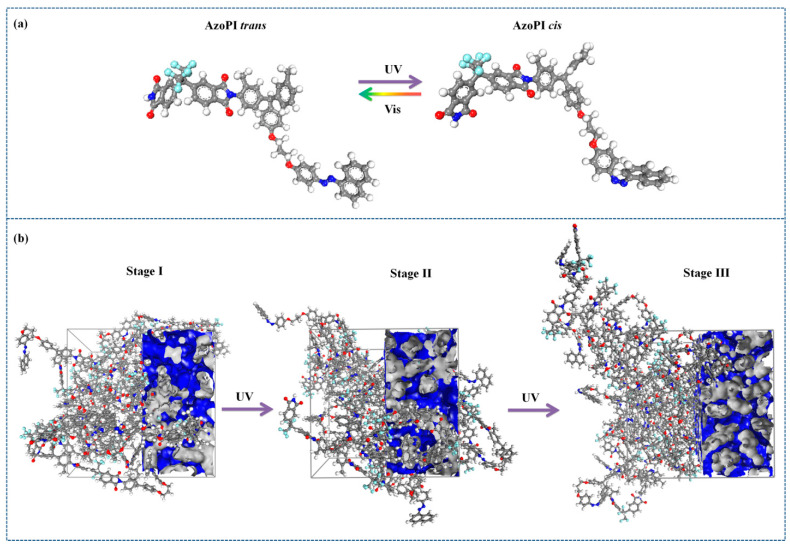
Molecular modeling of the photo-isomerization process of the azo-naphthalene groups: (**a**) minimum energy conformations of a AzoPI repeating unit in *trans* and *cis* states; (**b**) three-dimensional view of one amorphous cell for the AzoPI in stage I, stage II, and stage III (three polymer chains inside, each containing 10 repeating units; the grey surface indicates the Van der Waals surface, the blue surface indicates the void surface).

As mentioned above, the valley of the SRGs corresponds to the highest intensity of the UV laser irradiation through the phase mask. In this region, the macromolecules containing azo groups in the lateral chains undergo photo-isomerization during irradiation, when transitions from stable *trans* state to metastable *cis* state occurs along with changes in the molecular length and the dipole moment (Figure 9) [65]. This phenomenon will engender a localized substantial nanoscale stress, requiring more free volume for the local motion of azo groups from the lateral chain in *cis* configuration [52] and movement of the whole azo-polyimide backbone, inducing a dilatation effect and a continuous disturbance of the localized stress field [66].

Figure 9 shows three different stages of the system: stage I with polymer chains having azo-naphthalene modified in *cis* conformation in proportion of 15%, stage II with 50% azo- naphthalene groups in *cis* conformation, and stage III having all azo-naphthalene segments in *cis* form.

Molecular simulations have confirmed this expansion effect by higher values of the volume occupied by the polymer chains, V_o_, for the AzoPI in stage II compared to the stage I and the stage III (Table 3). Regardless of the mechanism involved in the photo-isomerization, rotation around the azo group, or inversion through one of the nitrogen nuclei, we found that a sufficiently large free volume was required to allow this mechanism, avoiding steric hindrances. It was found that the same system, AzoPI, had the largest free volume fraction in stage II. The free spaces between atoms provided the empty space useful for the movement of the molecular chains, and thus ensured the evolution towards the state III of the system.

In order to characterize the average configuration of a chain, we calculated the structural end to end distance (r_ee_) parameter. It was found that this parameter increased from stage I to stage II (Table 3) when the maximum value was reached, after which a decrease took place (stage III). In this case, first, the macromolecular chain stretched and then adopted a coil structure more compact than the starting one.

Cohesive energy density (CED) is a measure of the binding energy of the polymers relative to the unit volume. The CED also indicates the mixing degree of the polymer chains. Comparing the results from Table 3, we observed that the structure of AzoPI in stage II had the lowest cohesive energy density.

Saphiannikova et al. [67] demonstrated that the sign of the force induced by light is very sensitive to the molecular architecture, and therefore an effect of local extension occurs for amorphous azo-polymers. Consequently, due to the stress release in the UV laser-exposed region, a sudden modulating of the morphology takes place.

A very interesting phenomenon was observed. On SRG middle slopes, the average adhesion force and Young’s modulus were strongly reduced, simultaneous with the increases of the deformation (Table 2). Here, it seemed that the azo-material was softer, which may be related to a photo-induced reduction of the density [67,68,69]. Moreover, the molecular modeling data revealed that the structure with 50% of azo groups in *cis* conformation had the lowest value of cohesive energy density. With the decrease of the cohesive interactions, the force necessary to produce a deformation will decrease, meaning the decrease of Young’s modulus.

Moreover, it was observed that, although in the case of Azo-PI 45/100, when the number of pulses of irradiation was higher than those used in the Azo-PI 45/10 case, still the adhesion force and Young’s modulus were smaller. This is probably due to the higher number of *trans–cis–trans* photo-isomerization cycles of the azo-moieties that induce a material flow and implicitly a very weak plasticization. It is well understood that the material flow (as a dynamical process where material is moving) could not be directly measured here. However, plasticization could be inferred, with this being related to a change in the elastic properties (softening or hardening).

At the baseline, and especially on the bottom of new formed SRG, there was a high concentration of azo-naphthalene moieties in the *cis* state, which in both studied cases AzoPI 45/10 and AzoPI 45/100 changed the mechanical properties (see Table 2), slightly increasing the Young’s modulus, comparative to that obtained for the pristine sample. Hardening of the azo-polymer, as a result of an initial plasticization, was induced by the presence of a high population of *cis* fraction. The high density of *cis* isomers leads to an increase in the strength of intramolecular interactions in this region and also stronger interactions with the surrounding environment than *trans* isomers [70], a fact also proved by the CED values determined by the molecular dynamic simulations (Table 3). Thus, the local values of the Young’s modulus of the azo-polyimide are expected to vary with the concentration of *cis* isomers.

The rigidity, the hardening of the polymer, correlates at the microscale level with the mobility of the chains. The mean square displacement (MSD) function characterizes the movement of polymer chains by measuring the deviation of the position of a particle at a time (t) from the position it had at the reference time (0):

$$\mathrm{MSD}\;\left(t\right)={\left[r\left(t\right)-r\left(0\right)\right]}^{2}$$

**Figure 10:**
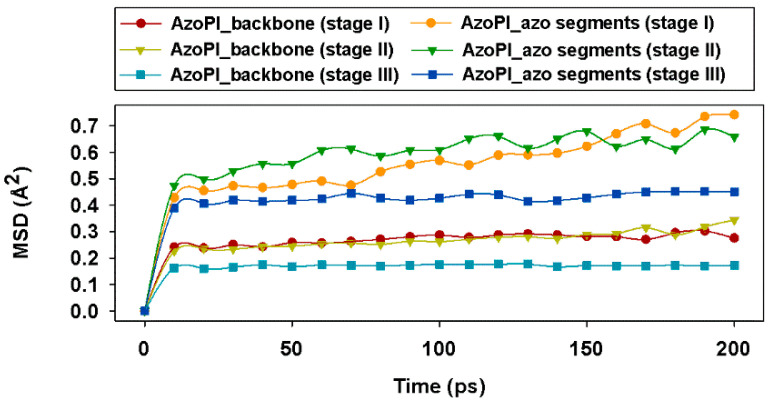
Evolution in time of the mean square displacement function of the AzoPI backbones and corresponding azo segments in stage I, stage II, and stage III.

According to Figure 10, the mobility of the backbone of AzoPI in stage III was the lowest, indicating that this system with all azo groups in the *cis* configuration was the most rigid. The intensity of the backbone movements for AzoPI in stage I and stage II were found to be similar over time. As expected, the azo segments (C–N=N–C) belonging to the polymer side chains had wider movements compared to the mobility of the main rigid chains. It was observed that the azo segments in the AzoPI in stage III chains mimicked the behavior of the chains from stage I, having much lower mobility compared to the other azo segments. The mobility of the azo segments belonging to the AzoPI polymers in stage I and II was approximately the same, reaching at 200 ps the segments connected to AzoPI in stage I to have a slightly higher motion than those connected to AzoPI in stage II.

**Figure 11:**
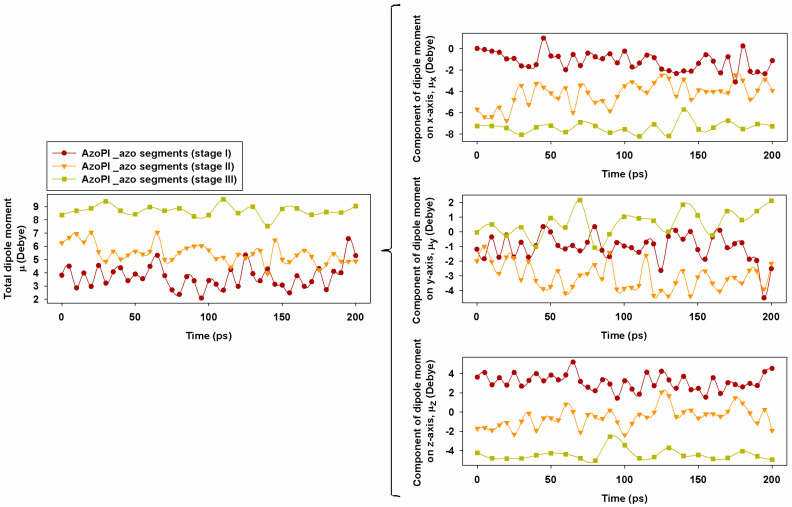
Evolution in time of total dipole moment (μ) and of the x, y, and z components of the dipole moment (μ_x_, μ_y_, and μ_z_, respectively) of the azo segments (C–N=N–C) of AzoPI in stage I, stage II, and stage III.

Moreover, the azo-naphthalene dipoles orientation can determine a more organized and condensed structure, having as a consequence the material contraction in the exposed regions, leading also to an increase of the hardness. If we consider the side azo segments (C–N=N–C) belonging to the three polymeric systems, the distribution of the dipole moment along the Cartesian axes as a function of time is represented graphically in Figure 11. However, we must specify that in the simulations the azo groups were left free to evolve, without any constraint of the dihedral angles.

The photoinduced molecular reorientations are those that dictate the direction and modulus of the dipole moment. The different values of the dipole moment obtained in our three considered cases indicate changes in the geometry and polarity of the azo segment over time. As can be seen, the dipole of the group defined by the azo segments was different from one stage to another, being dependent on the rigidity and geometry of the chain. On the other hand, the cumulative effect of lateral group movement can cause changes in the conformation of the molecule. By averaging the values of the dipole modulus along the 200 ps, we obtained the following values for the azo segments: μ_AzoPI_ = 3.7 D, μ_AzoPI_50%*cis*_ = 5.4 D, and μ_AzoPI_100%*cis*_ = 8.6, following the natural evolution of the modulus growth with the transition from the *trans* to the *cis* isomer.

As mentioned earlier, as a consequence of the interaction with the pulsed UV laser, during the *trans*–*cis* isomerization, the azo groups induce a very high pressure that determines the azo-polyimide to be able to develop high expansion forces during mass transport [52,71], which is liable for the irreversible deformations leading to SRG formation. In this stage, we found a certain state of matter, with a special feature, namely, extremely high viscosity and very low speed of polyimide chain displacement, on the strength of *trans–cis–trans* motion of azo-segments, that act as molecular motors [21]. The measurements performed on the peak of the sinusoidal pattern induced by this mass transport towards the lateral, together with the elastic material deformation determined by the supramolecular reorganization process, apparently indicated that no reaction takes place in this region (as a result of being protected by the mask). Thus, since these areas are rich in lateral chain azo groups in *trans* configuration, the values of the elastic modulus were close to that obtained for the pristine azo-polyimide (Table 2).

Essentially, during the dynamics of SRG formation process, there is a phenomenon in the variation of the material density [72].

### 3.3. Evaluation of the Local Chemical Properties

Raman spectroscopy was used to identify the signature of the aromatic azo group grafted on the polyimide chains before and after irradiation. According to literature data, the azo (-N=N-) stretching band (-N=N-) has strong intensity with the *trans* form absorbing in the range of 1465–1380 cm^−1^ and *cis* form around 1510 cm^−1^ [73,74].

**Figure A1:**
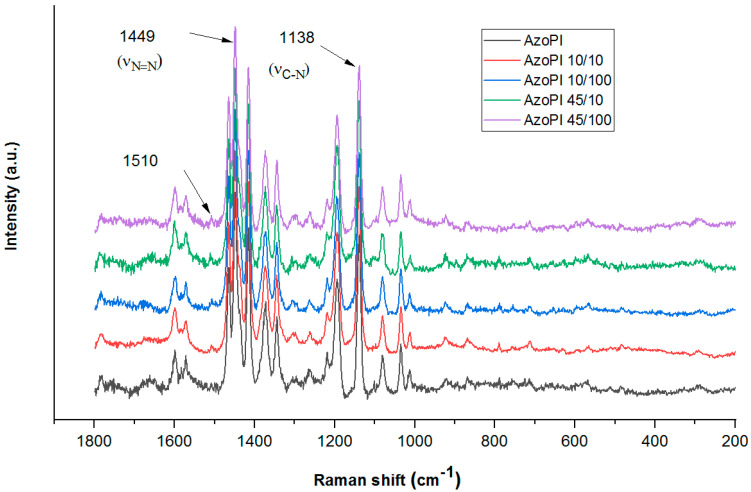
Raman spectra recorded on pristine and irradiated AzoPI film surfaces.

As can be seen in Figure A1, all the SRGs resulted after the treatment of azo-polyimide films with UV-pulsed laser radiation in different conditions showed the same strong absorption bands at 1449 cm^−1^ due to the distinct vibrational band (ν_N=N_) of the polymer chromophore that corresponded to the *trans* isomer. A very small absorption band around 1510 cm^−1^ could be observed as well for all irradiated samples, being associated with the vibrational band of the azo-group in *cis* form. It was obvious that this small absorption band slightly evolved with the increase of the incident fluence energy and number of pulses used for laser irradiation. Meanwhile, the vibrational band attributed to C–N bond (ν_C-N_) was identified for all azo-polyimide films at 1138 cm^−1^. Besides the appearance of the small absorption band around 1510 cm^−1^ associated with the vibrational band of the *cis* isomer formed after irradiation, the Raman spectra of irradiated films were not significantly altered as compared to that of the pristine sample, indicating that no chemical modification or degradation of the polyimide occurred during the irradiation. Accordingly, the changes detected in AFM measurements at the nanoscale were induced by conformational transitions, as also predicted by molecular modeling.

## 4. Conclusions

The aim of this study was to provide new insights regarding the formation mechanism of pulsed UV laser-nanoinduced patterns on azo-naphthalene-based polyimide films by evaluating the morphological, statistical, local mechanical, and chemical properties via AFM, in correlation with the molecular modeling. The quantitative nanomechanical properties (Young’s modulus, adhesion, deformation) of SRGs using the AFM PinPoint method were acquired with highly correlated topographic registration. The experimental evaluations highlighted different values of nanomechanical parameters obtained in different regions of the patterned relief. These were induced by re-organization of the matter by azo-naphthalene dipoles orientation and material photo-fluidization induced by repeated *trans*–*cis* isomerization of the azo-segments.

Attempts have been made to explain experimental phenomena by molecular modeling. The photoisomerization phenomenon was studied using three systems in which the content of *cis* isomers was gradually increased. Although the variations of the statistical and dynamic parameters such as the fractional free volume, end to end distance, mean square displacement, or dipole moment were small from one system to another, their evolution followed the same behavior as that observed at the macroscale during the photo-isomerization process. It was found that polymers with 50% azo groups in *cis* had either a maximum or a minimum peak of the calculated parameters. The low mobility of the chains with a maximum content in the *cis* isomer could explain the phenomenon of azopolymer hardening due to the photoisomerization process.

In addition, confocal Raman measurements evidenced no significant spectral changes, except for the slight increase of the absorption band due to the *cis* isomer evolution, demonstrating their origin in the polymer conformations before and after irradiation rather than in the polyimide chemical modifications.

## Figures and Tables

**Table 1:** 3D roughness parameters obtained from AFM images of the investigated azo-polyimide samples (energy density of 10 or 45 mJ/cm^2^ and variable number of laser pulses 10 or100).

Parameter	Sample			
	AzoPI 10/10	AzoPI 10/100	AzoPI 45/10	AzoPI 45/100
Height parameters				
Sq (nm)	0.4	0.732	16.159	28.723
Shape parameters				
Ssk	0.466	−0.284	0.762	0.343
Sku	5.466	3.061	2.354	3.193
Spatial parameters				
Stdi	0.388	0.418	0.193	0.407
Hybrid parameters				
Sdr (%)	0.00542	0.00981	1.496	5.342
Functional indexes				
Sbi	0.113	0.308	0.794	0.429
Sci	1.464	1.346	1.839	1.671
Svi	0.0855	0.131	0.046	0.096
Functional volume parameters				
Vmp (nm^3^/nm^2^)	0.0232	0.0242	0.562	1.62
Vmc (nm^3^/nm^2^)	0.365	0.664	13.300	25.4
Vvc (nm^3^/nm^2^)	0.463	0.875	25.400	36.6
Vvv (nm^3^/nm^2^)	0.0334	0.096	0.754	2.75

Sq: root mean square roughness of the surface; Ssk: skewness of height distribution; Sku: kurtosis of height distribution; Stdi: surface texture direction index; Sdr: surface area ratio; Sbi: surface bearing index; Sci: core fluid retention index; Svi: valley fluid retention index; Vmp: peak material volume; Vmc: core material volume; Vvc: core void volume; Vvv: valley void volume.

**Table 2:** The values of the nanomechanical characteristics (adhesion force, deformation, and Young’s modulus) measured at different positions on the azo-polyimide samples before and after laser irradiation by using AFM PinPoint mode.

Sample	Position on the Sample	Nanomechanical Characteristics		
		Adhesion Force (nN)	Deformation (nm)	Young’s Modulus (MPa)
AzoPI	All over the surface	12.6 ± 0.8	5.1 ± 0.2	294.8 ± 20.5
AzoPI 45/10	SRG top hills	11.6 ± 0.3	6.3 ± 0.1	299.8 ± 5.5
	SRG middle slopes	6.2 ± 0.7	8.9 ± 0.1	195.1 ± 4.6
	SRG bottom valleys	12.1 ± 0.1	6.7 ± 0.1	311.0 ± 5.2
	SRG base line	11.4 ± 0.4	6.8 ± 0.2	302.8 ± 9.1
AzoPI 45/100	SRG top hills	12.9 ± 1.9	5.4 ± 0.2	294.2 ± 9.0
	SRG middle slopes	3.2 ± 0.4	8.9 ± 0.3	128.9 ± 8.6
	SRG bottom valleys	13.3 ± 0.1	5.1 ± 0.2	307.9 ± 12.2
	SRG base line	12.4 ± 0.3	5.1 ± 0.1	301.9 ± 12.3

**Table 3:** Predicted parameters for the azo-polyimide systems studied by molecular simulation.

AzoPI Sample	Parameter					
	ρ_p_ (g/cm^3^)	V_o_ (Å^3^)	V_f_ (Å^3^)	FFV	r_ee_ (Å)	CED × 10^7^ (cal/m^3^)
Stage I	1.20	25,012	17,105	40.61	31.06	5.8409
Stage II	1.19	25,025	17,642	41.35	36.55	5.7983
Stage III	1.21	24,993	16,649	39.98	28.95	6.0190

ρ_p_—density of packing; V_o_—occupied volume with the atoms being represented by Van der Waals radii; V_f_—free volume; FFV = (V_f/_(V_o_ + V_f_))∙100—fractional free volume; r_ee_—end-to-end distance of the polymers; CED—cohesive energy density.

## Data Availability

The data is available on the request from corresponding author.

## Appendix A

The use of two 3D topographic parameters, namely, surface skewness (Ssk) and coefficient of kurtosis (Sku), allowed us to measure the departure from a Gaussian distribution of surface heights, emphasizing on one hand the degree of symmetry of the surface heights reported to the mean plane (Ssk), and on another hand the spread of the newly created structures in relation with the whole surface morphology (Sku). Consequently, the value of the skewness was influenced by the surface dislevelment, which can be above (negative skewed) or below (positive skewed) the mean surface height, implicitly being sensitive to occasional deep valleys or high peaks. The negative skewness obtained for AzoPI 10/100 sample (Table 1, Figure 4c) indicated surfaces with truncated peaks or occasional deep valleys (as seen in the cross-section profiles from Figure 3d). The positive skewness, calculated for AzoPI 10/10, AzoPI 45/10, and AzoPI 45/100 (Table 1, Figure 4a,e,g), revealed profiles with valleys filled in, or occasional high peaks, as pictured in Figure 3b,f,h by the cross-section profiles. The lower positive value of the kurtosis (Sku < 3) obtained for the homogeneous periodic AzoPI 45/10 sample (Table 1) indicated that the surface was platykurtic (Figure 4e), presenting a bumpy morphology with a relatively even distribution of heights above and below the mean height (as seen in Figure 3f of the cross-section profile). Thus, the valley zones and the up hills were well-defined. The higher positive values of the kurtosis (Sku > 3) obtained for AzoPI 10/10, AzoPI 10/100, and AzoPI 45/100 (Table 1) suggest leptokurtic surfaces (Figure 4a,c,g), with surface relief gratings with spiky morphologies (as observed also from the cross-section profiles from Figure 3b,d,h).

## Appendix B

Along with 3D imaging at the nanoscale, nanomechanical properties (including adhesion, hardness, etc.) measurement on the sample surface at nN-scale via force–distance (F-d) spectroscopy by using a cantilever tip to probe the sample is one of the most well-known functions of AFM. This technique, widely used in domains such as physics, chemistry, and materials science dealing with nanoscale research, generally implies firstly acquiring a reference map of the topography data of a sample, on which the force–distance measurements are subsequently be made in the regions of interest after a preliminary alignment. Still, it is hard to prevent both positional errors in force–distance spectroscopy data and topographical data, as well as the artifacts induced by the adhesive tip–soft sample interaction. This is why PinPoint Nanomechanical mode was designed to prevent these positional errors by simultaneously acquiring accurate height and force–distance information in each of 256 × 256 pixels in the whole scanning area, while moreover preventing the cantilever tip from grabbing additional material from the surface when it travels to the next pixel position by lifting it at every pixel at a perpendicular angle from the sample surface. For this, some preset parameters are necessary. One of the most important is the control height, which is determined in the AFM cantilever retraction process from the sample surface in its movement between pixels and is dependent on the features of the sample surface. For example, if the surface heights are small, the control height must be small, and vice versa. However, in some cases, if the surface sample has small heights, but is tacky, then the height control must be suitably enlarged in order to separate the tip from the sample in anticipation of the switch to the next pixel. Other necessary preset parameters are the stiffness threshold (which establishes the force frequently acting on the surface sample), the approach and retract time, the XY pixel-to-pixel movement time, the pre-approach delay for determining a scanning speed, and the lift height. Thus, an optimized surface morphology providing precisely the height information is determined.

In the first instance, PinPoint mode imaging can generate an adhesion map using the maximum adhesion force value by acquiring in each pixel the adhesion force (*F*_adh_) between the structured formations and silicon cantilever, calculated from each force–distance curve (as the one from Figure 5) as a linear function of the probe displacement relative to the sample surface along to the *Z*-axis, according to Hooke’s law, expressed by

$$F_{\mathrm{adh}}=-k\Delta x,$$

where *k* is cantilever stiffness and Δ*x* is deflection of the cantilever in rapport with azo-polyimide SRG.

The sample’s stiffness/elasticity (Young’s modulus) can be acquired, similar to the force–distance spectroscopy procedure, from the slope of the force–distance curve taken at each pixel, from the point of contact to the deflection threshold, as seen in Figure 5, fitted with the Hertz model [59,60,61]. Assuming that no friction exists between the azo-polyimide sample and the elastic sphere tip with radius R, which indents to a displacement d, the applied force can be derived as (Figure 5)

$$F_{\mathrm{appl}}=\frac{4}{3}E^{*}\sqrt{Rd^{3}},$$

where *E** is the effective elastic modulus. *E** can be calculated by measuring *F*_appl_, *R*, and *d*:

$$E^{*}=\frac{3}{4}\frac{F_{\mathrm{appl}}}{\sqrt{Rd^{3}}}$$

In order to evaluate the deformation depth from the surface of the sample, *d*, one must change the force–distance curve to a tip-sample separation. At the same time, *E** is a function of the two materials implied in the interaction, namely, the one of the tip and the one of the sample:

$$\frac{1}{E^{*}}=\frac{1-\nu_{\mathrm{tip}}^{2}}{E_{\mathrm{tip}}}+\frac{1-\nu_{\mathrm{sample}}^{2}}{E_{\mathrm{sample}}}$$

where *E*_tip_ is the elastic modulus of the tip, *E*_sample_ is the elastic modulus of the sample, *ν*_tip_ is the Poisson’s ratio of the tip, and *ν*_sample_ is the Poisson’s ratio of the sample. The Poisson’s ratio for the Si AFM tip was set at 0.27 [62] and for azo-polyimide sample was set at 0.35 [63,64]. Knowing *E**, *E*_tip_, *ν*_tip_, and *ν*_sample_, *E*_sample_ can be back-calculated with the formula

$$E_{\mathrm{sample}}=\frac{1-\nu_{\mathrm{sample}}^{2}}{\frac{1}{E^{*}}-\frac{1-\nu_{\mathrm{tip}}^{2}}{E_{\mathrm{tip}}}}$$

In this way, the map of the sample’s stiffness/elasticity can be simultaneously acquired through the adhesion force map with the topographic sample information, locally resolving the distribution of adhesion and elasticity on the investigated surface.
